# Naltrexone and Nalmefene as Modern Psychopharmacotherapy for Alcohol Use Disorder: Modulation of Opioid Receptors and Neurobiological Pathways of Alcohol Action

**DOI:** 10.3390/biomedicines14061356

**Published:** 2026-06-16

**Authors:** Maciej Rząca, Mateusz Sroka, Katarzyna Fus, Dawid Ślebioda, Rozalia Kozinska, Mateusz Chmiela, Agnieszka Chłopaś-Konowałek

**Affiliations:** 1 Scientific Society for Neurotoxicology, Department of Forensic Medicine, Wroclaw Medical University, Mikulicza-Radeckiego 4J, 50-345 Wroclaw, Poland; maciej.rzaca@student.umw.edu.pl (M.R.); mateusz.sroka@student.umw.edu.pl (M.S.); katarzyna.fus@student.umw.edu.pl (K.F.); dawid.slebioda@student.umw.edu.pl (D.Ś.); 2 Scientific Society for Psychopharmacology, Department of Forensic Medicine, Wroclaw Medical University, Mikulicza-Radeckiego 4J, 50-345 Wroclaw, Polandmateusz.chmiela@student.umw.edu.pl (M.C.); 3 Department of Forensic Medicine, Division of Molecular Techniques, Wroclaw Medical University, Sklodowskiej-Curie 52, 50-369 Wroclaw, Poland

**Keywords:** alcohol use disorder (AUD), addiction, pharmacological interventions, naltrexone, nalmefene

## Abstract

**Background:** Alcohol use disorder (AUD) is a grave mental health condition that can result in significant health and social consequences. The medications Naltrexone and Nalmefene are indicated for the treatment of AUD, with Naltrexone having received the most extensive research attention. **Methods:** The majority of papers assessing universal measures of alcohol consumption employed two primary metrics: total alcohol consumption (TAC) and the number of days per month where individuals engaged in heavy drinking (HDD). Indicators pertaining to the maintenance of complete abstinence were excluded due to the absence of sufficient data. The safety of both substances was also assessed, as were the frequency of side effects and independent patient dropout. The study also incorporated practical factors of the therapy, such as the route of administration, dosage regimen, and the drug’s patient convenience, which can have a significant impact on adherence to therapy. **Results:** Nalmefene, administered in an “as needed” regimen, demonstrated statistically significant activity in reducing HDD and total alcohol consumption (TAC) among patients with AUD, particularly those with elevated World Health Organization (WHO) DRL risk. Preliminary findings from the ESENSE1 (Efficacy of Nalmefene in Alcohol Dependence; the first phase III study), ESENSE 2 (Efficacy of Nalmefene in Alcohol Dependence, the second phase III study), and SENSE (the final phase III long term-safety and cost-effectiveness study) studies indicate a substantial decrease in HDD and TAC following the initial month of treatment. These effects persist throughout the subsequent follow-up period. Several Japanese studies have corroborated the effectiveness of Nalmefene, demonstrating its efficacy across both short-term and long-term applications. Furthermore, these studies have substantiated its safety profile, indicating that there is no inherent risk of addiction or the emergence of withdrawal symptoms. The mild nature of adverse events (most commonly nausea and dizziness) led to a relatively low discontinuation rate of Nalmefene treatment. A subsequent study, employing a recognized methodology, corroborated the efficacy of psychosocial support in enhancing treatment outcomes. Meta-analyses demonstrate that Naltrexone exhibits comparable efficacy in reducing the frequency and severity of alcohol consumption. In select populations, the injectable form (LAI) of this pharmaceutical agent facilitates less frequent dosing, which is advantageous for the treatment process. A comparison of Nalmefene and Naltrexone reveals that the latter does not demonstrate a significant impact on the likelihood of individuals returning to heavy alcohol consumption. **Conclusions:** In the treatment of AUD, both naltrexone and nalmefene have been shown to yield positive outcomes, particularly in terms of reducing the HDD and TAC. According to the World Health Organization (WHO) classification, Nalmefene is indicated for individuals with a high risk of developing serious conditions. It has been demonstrated to produce rapid and sustained results while exhibiting a favorable safety profile, characterized by the absence of significant adverse effects. Naltrexone is a medication that has proven to be effective. LAI may have a positive impact on the efficacy of treatment.

## 1. Introduction

Alcohol has played a significant role in human social life for millennia, fostering relationships and integration between individuals. The consumption of alcohol is a primary source of pleasure for humans. Alcohol consumption, even in its initial stages, has been demonstrated to induce alterations in neural circuits, which can be perceived as a means of alleviating anxiety and eliciting pleasure. These effects manifest as feelings of euphoria and relaxation that can ensue from alcohol consumption [1]. The consumption of alcoholic beverages, particularly in excess, can result in a multitude of adverse consequences, including an elevated risk of health complications, an increase in criminal activity, road traffic incidents, and, in certain instances, the development of alcohol use disorder (AUD) [2].

### 1.1. Alcohol Use Disorder

AUD is a major mental health problem that can lead to serious health and social consequences. AUD can be a chronic condition characterized by behavioral and physiological changes observed in people who chronically consume alcohol. The diagnosis of AUD may be based on tolerance to the effects of alcohol, craving, hazardous use, and a significant amount of time spent drinking alcoholic beverages [3].

According to the 2018 World Report on Alcohol and Health by the World Health Organization (WHO), in 2016, excessive alcohol consumption was responsible for approximately 3 million deaths worldwide, constituting 5.3% of all deaths reported. This figure exceeds the combined total of deaths attributable to hypertension and diabetes. According to estimates from the aforementioned report, alcohol consumption contributed 5.1% to the global burden of disease and injury, resulting in 132.6 million disability-adjusted life years (DALYs). In 2016, approximately 2.3 billion individuals consumed alcohol, and the number of individuals with AUD over the age of 15 was 283 million, accounting for 5.1% of the adult population. In middle- and high-income countries, the economic costs associated with alcohol consumption were estimated at more than 1% of gross national product. Alcohol was the seventh most common factor associated with the risk of premature death and disability. According to the WHO, while per capita alcohol consumption has declined in some regions, projections indicate an increase in global alcohol consumption over the next decade, which could result in a higher health burden [4]. According to data from the WHO pertaining to the year 2019, the consumption of alcohol in excess had a significant impact on the global burden of disease, accounting for 6.9% of cases in men and 2.0% in women. Alcohol, a pivotal risk factor for premature death and disability within the 20 to 39 age group, was responsible for 13% of all deaths among individuals in that age range. Individuals in vulnerable categories exhibited notably elevated rates of alcohol-related mortality and hospitalization [5].

### 1.2. Alcohol Consumption and Suicide Risk

Alcohol consumption exerts a substantial influence on the phenomenon of suicide across its various stages, ranging from the initial development of suicidal ideation to the ultimate act of completed suicide. Indeed, the consumption of alcohol in excess is the second strongest risk factor for suicide, exceeded only by affective disorders such as depression [5]. The suicide rate from 2003 to 2018 exhibited a substantial annual increase among women across all age groups: the young (2.8%; 95% CI: 1.86–3.75%), the middle-aged (2.20%; 95% CI: 1.20–3.21%), and the elderly (10.48%; 95% CI: 1.17–20.65%). A substantial increase was observed exclusively among middle-aged men (0.81%; 95% CI: 0.003–1.62%) [6]. Guo et al. found that a 1 L increase in alcohol per capita (APC) at the population level was associated with a 3.59% increase in suicide mortality [7]. On the other hand, Isaacs et al. demonstrated that alcohol consumption increased the risk of death by suicide by 94% [8].

### 1.3. Classification of Risks Associated with Alcohol Consumption

In the case of the male population, very high-risk drinking is defined as drinking more than 100 g per day, or more than 7.1 standard drinks in the United States. High risk is indicated when alcohol consumption ranges from 60 to 100 g per day, equivalent to 4.3 to 7.1 drinks. Moderate risk is associated with alcohol consumption ranging from 40 to 60 g per day, equivalent to 2.9 to 4.3 drinks. Finally, low-risk consumption is defined as intake ranging from 1 to 40 g per day, equivalent to 1 to 2.9 drinks [9].

For women, very high risk is defined as alcohol consumption of more than 60 g per day, or more than 4.3 drinks. High risk is indicated by alcohol consumption ranging from 40 to 60 g per day, equivalent to 2.9 to 4.3 drinks. Moderate risk is associated with alcohol consumption ranging from 20 to 40 g per day, which is equivalent to 1.4 to 2.9 drinks. Finally, low-risk consumption is defined as alcohol intake ranging from 1 to 20 g per day, equivalent to 1 to 1.4 drinks [10].

### 1.4. Characteristics of Alcohol Addiction

Alcohol addiction is a chronic disease marked by recurrent episodes of relapse and remission. According to the Diagnostic and Statistical Manual of Mental Disorders, 5th edition (DSM-5), AUD is characterized by a series of neurobiological alterations and maladaptive behaviors, including the development of tolerance, withdrawal symptoms, escalating alcohol consumption, and a strong desire to drink. The development of addiction to a variety of substances, including alcohol, results in the constant seeking of alcohol and the persistence of addiction, where a number of neurotransmitters are involved, such as serotonin, dopamine, opioid peptides, glutamate, and γ-aminobutyric acid (GABA) [11].

### 1.5. Role of Dopamine in Alcohol Addiction

Dopamine has been implicated in the cycle of alcohol abuse. The regulation of the reward system is partially mediated by the dopaminergic mesolimbic pathway, which originates in the ventral tegmental area (VTA). The projections from this area involve the striatum and the prefrontal cortex. The reward system plays a pivotal role in shaping goal-focused behavior through reinforcement mechanisms. It responds to rewarding stimuli, including food, money, and addictive substances. Psychoactive substances, such as alcohol, have been shown to induce neurobiological changes that modify the mechanisms responsible for reward sensation, behavior, and motivation by affecting the reward system [12].

### 1.6. Genetic and Environmental Factors in AUD

A decline in dopamine receptor numbers is frequently attributed to genetic and environmental factors, particularly in individuals who experienced neglect in early childhood. This decline can result in a diminished capacity to experience pleasure from particular substances. This phenomenon can lead to an escalation in alcohol consumption, which is a major public health concern [13]. A genetic predisposition, in consideration of a family history of AUD, results in an increased likelihood of developing the condition. Furthermore, evidence suggests that adopted children whose biological parents are affected by AUD may face an elevated risk of developing the condition themselves. Studies of twins have indicated that the heritability of AUD can range from 50% to 60%, with higher values observed in monozygotic twins. Furthermore, studies have identified the GDAP1 gene as a potential contributor to AUD, reporting abnormal DNA methylation and histone modifications in individuals with a history of the condition. This disruption may contribute to the development of addiction by influencing neuronal pathways. According to neurochemical studies, one of the most significant changes associated with alcoholism is the increased expression of the glutamate transporter GLAST/EAAT1 in the brain. GLAST/EAAT1 is a highly abundant protein in neural tissue and is closely associated with the functioning of excitatory (glutamatergic) synapses. Alterations in its expression may represent a significant mechanism affecting brain function in individuals with chronic alcohol abuse [14,15].

### 1.7. Synaptic Plasticity and Its Role in Addiction

The synaptic plasticity, which underlies learning processes, has been identified in neural reward circuits and has been demonstrated to significantly influence the development of addictive behaviors, both in the context of alcohol and drugs. Chronic exposure to addictive substances, such as alcohol, has been demonstrated to result in the formation of enduring memories associated with the experience of using them. The treatment of addiction relapse constitutes a significant clinical challenge, frequently resulting from exposure to stimuli associated with the experience of addiction. The consequences of protracted exposure to alcohol and other addictive substances manifest as persistent forms of synaptic plasticity, which are associated with certain behavioral effects and challenges in maintaining abstinence [16].

### 1.8. Functional Consequences of Alcohol Consumption

Alcohol consumption has been associated with many functional consequences, including decreased cognitive flexibility, reduced behavioral efficiency, and increased anxiety. Furthermore, alcohol has been demonstrated to facilitate the dissolution of inhibitions, amplify impulsivity, and engender a proclivity for risk-taking behaviors [9].

## 2. Effect of Ethanol on Neurotransmission

### 2.1. GABAergic System

GABA is a pivotal neurotransmitter that exerts inhibitory effects within the central nervous system (CNS). Its signaling transpires through interactions with two primary types of receptors: GABA_A_ and GABA_B_. GABA_A_ receptors function as ionotropic chloride channels and mediate rapid inhibitory signaling, whereas GABA_B_ receptors are metabotropic G protein-coupled receptors (GPCRs) involved in slow inhibition [17].

The GABA_A_ receptors are ionotropic receptors present throughout the brain, including areas involved in alcohol use, such as the prefrontal cortex, thalamus, cerebellum, and amygdala [17,18]. These receptors are classified as heteropentamers, comprising a combination of 19 distinct subunits: six α (alpha1-6), three β (beta1-3), three γ (gamma1-3), three ρ (rho1-3), and one each of δ (delta), ε (epsilon), π (pi), and θ (θeta). The result of this process is the formation of a significant number of receptor isoforms. However, the most prevalent form of the GABA_A_ receptor consists of two α subunits, two β subunits, and one γ subunit arranged as γ_2_β_2_α_1_β_2_α_1_ counterclockwise around the center [19]. The binding of ethanol to GABA_A_ receptors involves interactions with multiple subunits, particularly the α subunit [17]. A role for ethanol in the expression and transport of GABA_A_ receptors is evident even at low levels of ethanol, as evidenced by a rapid reduction in the expression of α_4_β_3_δ-GABA_A_ receptors in the hippocampus. Furthermore, the expression of α_1_β_3_γ_2_—GABA_A_ receptors also decreases a few hours after ingestion. Conversely, the expression of α_4_β_3_γ_2_ and α_2_β_3_γ_1_ increases after several days [17]. This mechanism of action is referred to as a positive allosteric modulator (PAM), and classifies ethanol’s properties as sedative and neuromodulatory [17]. At low levels of alcohol consumption, corresponding to blood alcohol concentrations of 0.02–0.04 g/dL, the enhanced action of GABA through its effect on GABA_A_ receptors induces relaxation. In the case of moderate-risk drinking episodes, that is, at ethanol levels <0.08 g/dL, there is an enhancement of GABA_A_ receptor activity in the brain, leading to a decrease in excitatory glutamatergic neurotransmission. The effect is characterized by mild sedation, a sense of relief, minor alterations in short-term memory, and diminished attention. The consumption of alcohol that results in a blood alcohol concentration > 0.08 g/dL is classified as acute, and it is characterized by the presence of cognitive deficits and a diminished capacity to voluntarily regulate alcohol intake. This has been demonstrated to result in an elevated risk of developing AUD in the future [17].

**Figure 1:**
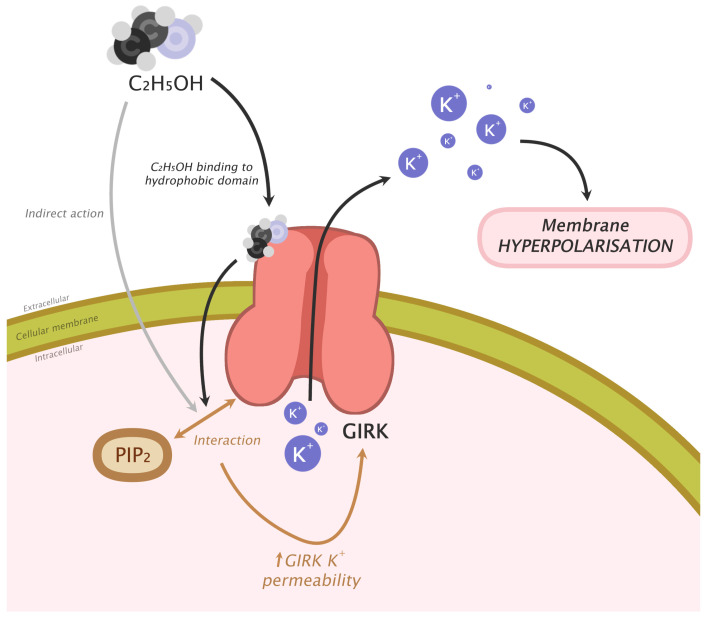
Hyperpolarization of the cell membrane and a decrease in neuronal excitability. Original concept by Ratka, Z.; Final draft by Wolak K. Legend: Grey arrow—indirect action; brown arrow—interaction; black arrow—direction in which molecules go.

The GABA_B_ receptors are metabotropic G-protein-coupled receptors located at presynaptic, postsynaptic, and extrasynaptic sites, showing a wide distribution in both the central and peripheral nervous systems. Their distribution encompasses brain structures involved in the regulation of alcohol-related behavior, such as the VTA, the amygdala, and the globus pallidus [20,21,22]. The GABA_B_Rs are obligate heterodimers, composed of GABA_B1_ and GABA_B2_ subunits. Each subunit possesses an extracellular VFT domain, which is linked to the heptahelical transmembrane domain (7TM) by a short linker that lacks cysteine residues [21]. Laboratory studies in mice have demonstrated that ethanol exerts an enhancing effect on signaling through GABA_B_, likely through its interaction with and activation of its primary somatodendritic effector, the G-protein-gated K^+^ and inwardly rectifying (GIRK) channel [20]. The effect of ethanol on GIRK channels is accomplished by directly binding to a hydrophobic pocket in the channel structure, thereby increasing its interaction with phosphatidylinositol-4,5-bisphosphate (PIP_2_). The activated channel increases potassium ion (K^+^) efflux from the cell, hyperpolarizing the membrane and reducing neuronal excitability. Furthermore, ethanol, in addition to its direct binding to GIRK channels, has the capacity to modulate their activity by altering their affinity for PIP_2_ [18]. The aforementioned mechanism is illustrated in Figure 1.

**Figure 2:**
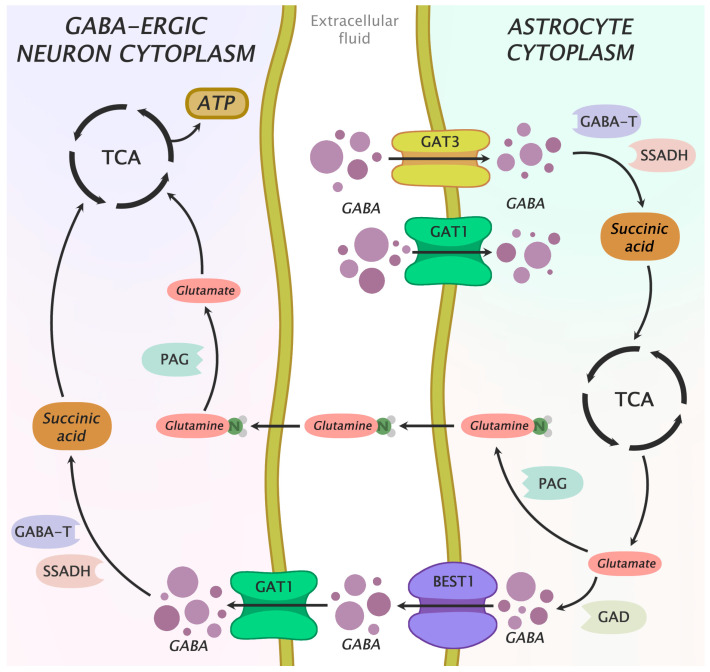
Regulation and maintenance of GABA levels in the brain. Original concept by Ratka, Z.; Final draft by Wolak K. Legend: smooth arrow—direct reaction; bold arrow—cycles of reactions.

Recent studies have demonstrated that alterations in GABA transmission predominantly impact regions implicated in the negative reinforcing effects of alcohol. These regions encompass the amygdala, the globus pallidus, and the VTA [23]. The regulation and maintenance of GABA levels in the brain are primarily influenced by interactions between astrocytes and neurons, as illustrated in Figure 2. The process of converting glutamate into GABA, a critical neurotransmitter, is initiated by the enzyme phospho-activated glutaminase (PAG), produced by astrocytes and subsequently utilized by neurons. Conversely, astrocytes play a pivotal role in the reverse process of converting glutamate back into glutamine. However, they can also synthesize GABA from glutamate via a glutamate decarboxylase (GAD)—dependent pathway. The sequential release of GABA from the synapse is facilitated by bestrophin 1 (BEST1)—mediated channels, and its excess is subsequently taken up by GAT1 and GAT3 transporters in astrocytes and GAT1 in GABAergic neurons. The captured GABA is subsequently degraded by the enzymes GABA-transaminase (GABA-T) and succinate semialdehyde dehydrogenase (SSADH), which act on the intermediate product, succinate semialdehyde, as previously documented [23].

GABA, a major inhibitory neurotransmitter, is released from astrocytes into the extracellular space via three distinct mechanisms. These mechanisms include GABA-permeable channels, calcium-dependent vesicular exocytosis, and GABA transporters (GATs). GATs are classified as secondary, active, electrogenic transporters. The transport process entails the exchange of sodium and chloride ions with GABA uptake. The GATs are classified into four primary types: GAT1, GAT2, GAT3, and GAT4. The GAT1 and the GAT3 exhibit a high affinity for GABA [22]. Recent studies on astrocyte GABAergic signaling have revealed its key role in cognitive function and behavioral regulation [23].

A study by Mederos et al. utilized optogenetic and behavioral techniques on adult laboratory mice [24]. The study demonstrated that the genetic ablation of GABA_B_ receptors in astrocytes of the central prefrontal cortex led to alterations in low-frequency gamma oscillations and changes in the properties of neuronal discharges in the cerebral cortex. These changes, in turn, affected processes related to goal-directed behavior [21]. The statistical analyses conducted on the study’s results demonstrated statistically significant differences in the behavior of mice at a level of *p* < 0.05, following manipulations of GABAergic signaling to astrocytes, in comparison to the control group, i.e., mice not subjected to optogenetic manipulations [24].

A study by Kang et al. [25] used chemogenetic activation of astrocytes in the dorsomedial striatum (DMS) of 8–10-week-old male mice, which was confirmed by calcium imaging [25]. This activation was found to differentially modulate the activity of medial striatal spiny neurons (MNS), resulting in a shift from habitual to goal-directed behavior during reward seeking [23]. Statistical analyses with a significance level of *p* < 0.05 revealed statistically significant changes in neuronal synaptic activity and reward-seeking behavior in the astrocyte activation group compared to the control group, i.e., mice not subjected to astrocyte activation [25].

In a separate study by Kang et al. [26], researchers employed chemogenetic techniques to selectively activate astrocytes in the external globus pallidus (GPe) of 8- to 10-week-old male mice. This activation was confirmed through calcium imaging [26]. The study observed that astrocyte activation leads to a decline in GPe neuronal activity, which in turn promotes the transition from habitual to goal-directed alcohol-seeking behavior. During the process of habit formation, an increase in GAT3 transporter mRNA levels has been detected in the GPe by quantitative real-time PCR [26]. Inhibition of GAT3 activity through selective means resulted in the reversal of astrocyte-induced alterations in neuronal activity, thereby affecting the transition from habitual to goal-directed alcohol-seeking behavior. The results demonstrated statistically significant alterations in reward-seeking behavior after astrocyte activation in GPe, in comparison to the control group, i.e., mice injected with the control vector. This finding was confirmed by statistical analyses with a significance level of *p* < 0.05 [23,26]. This finding suggests that increased GAT3 expression in the GPe may result in astrocyte deactivation, thereby attenuating its inhibitory effects on GPe neurons. This study underscores the critical role of astrocytic GAT3 in regulating reward-related behavior in the GPe [23].

A study by Augier et al. employed behavioral techniques and quantitative real-time PCR (qPCR) and AAV viral vectors [27]. This enabled the demonstration of reduced levels of mRNA and GAT3 in the amygdala following excessive alcohol stimulation. The amygdala, a forebrain structure, is recognized as the central hub of GABAergic influences on alcohol reinforcement. The observed decrease in mRNA and GAT3 following ethanol stimulation was found to be associated with a concurrent decrease in GABA_A_R subunits. This finding supports the hypothesis that an increase in these subunits is associated with increased GABAergic system activity, resulting from reduced extracellular GABA clearance [23]. The results demonstrated statistically significant differences in alcohol choice behavior after manipulations of GAT3 expression, compared to the control group, i.e., rats injected with the control vector and rats not subjected to genetic manipulation. These outcomes were corroborated by statistical analyses with a significance level of *p* < 0.05 [27].

### 2.2. The Glutamatergic System

The N-methyl-D-aspartate receptor (NMDAR) is a critical ionotropic receptor implicated in various aspects of brain development, synaptic plasticity, and cognitive functions, including learning and memory [28].

**Figure 3:**
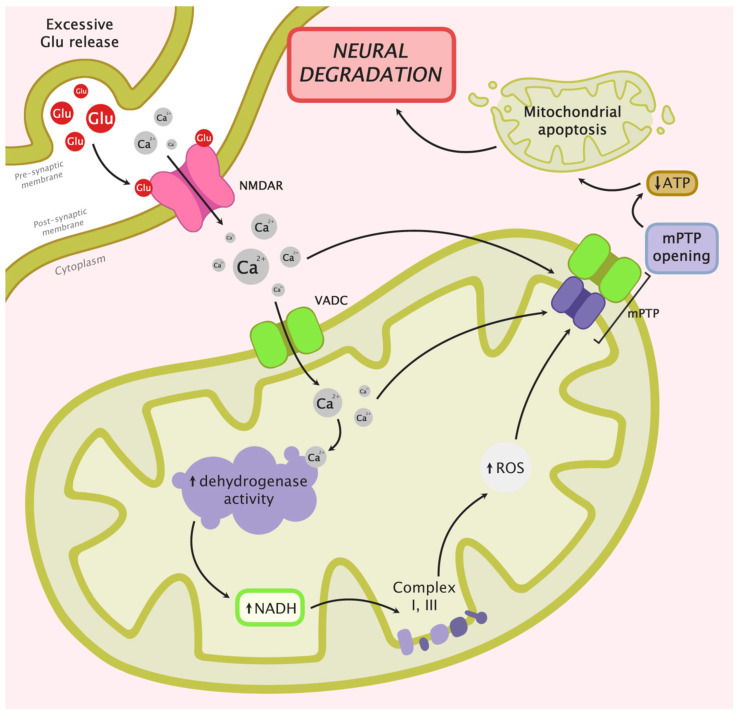
Initiation of signaling pathways that are responsible for mitochondrial apoptosis. Original concept by Ratka, Z.; Final draft by Wolak K. Legend: arrow—direction of reactions.

The neurotransmitter that activates NMDARs is glutamate, which plays a major excitatory role in the central nervous system. For full NMDAR activation upon glutamate binding, the presence of glycine or D-serine is essential, acting as co-agonists. Conversely, the receptor undergoes a blockade in a voltage-dependent manner through Mg^2+^. The result of this process is the flow of calcium and sodium ions into the neuron, leading to depolarization of the cell [28,29]. A multitude of effects have been identified in studies on animal models, including rats and laboratory mice, arising from ethanol’s action on the NMDA receptor, its primary site of action in the brain. These include ethanol-induced excitotoxicity and impaired synaptic plasticity [28]. The NMDA receptors interact selectively with ethanol and are an important neurochemical component of addiction and withdrawal. This dysregulation arises from long-term alcohol abuse. Its underlying mechanism is the disruption of glutamatergic homeostasis within NMDA receptors. The result of prolonged ethanol exposure is inhibition of glutamatergic transmission, which is compensated by increases in the number, density, and composition of NMDA receptors at the cellular and synaptic levels. The synthesis and incorporation of additional receptors into synapses, as well as the recruitment of receptors from the intracellular pool or the local extracellular region, have been observed. However, when ethanol is eliminated from the system during withdrawal, its inhibitory effect disappears, contributing to over-stimulation of NMDA receptors and subsequent excitotoxic hyperactivity. This over-stimulation is a factor for excitotoxic cell death, as well as delayed neuronal degeneration, manifesting as withdrawal seizures [30]. An increase in glutamate concentration to millimolar values in the synaptic gap has been shown to cause excessive activation of postsynaptic glutamate receptors. The heightened stimulation of these receptors determines the substantial influx of Ca^2+^ and, via voltage-gated VDAC channels, the influx of these ions into the mitochondria. The increased mitochondrial Ca^2+^ then stimulates the activity of mitochondrial dehydrogenase enzymes, leading to the synthesis of NADH, which functions as the electron (e^−^) donor for the electron transport chain (ETC). The release of electrons from complexes I and III results in an incomplete reduction of O_2_, leading to the formation of reactive oxygen species (ROS). The accumulation of Ca^2+^ in both the mitochondria and the cytoplasm, in conjunction with elevated ROS production, disrupts mitochondrial membrane stability, culminating in the opening of mitochondrial permeability transition pores (mPTPs). The subsequent activation of these pores leads to ATP depletion and the initiation of signaling pathways that mediate mitochondrial apoptosis, resulting in neuronal damage [31]. The aforementioned mechanism is illustrated in Figure 3. Furthermore, a multitude of phenomena are commonly associated with ethanol, arising from its impact on NMDA receptors. These include alcohol craving, increased tolerance to alcohol, alcohol dependence, and alcohol withdrawal syndrome (Delirium tremens, DT) [32].

The NMDA receptor is classified as an ionotropic cationic receptor, exhibiting a high affinity for glutamate and utilizing glycine or D-serine as co-agonists. NMDAR is a heterotetramer composed of two obligate GluN1 subunits and two GluN2 subunits, having four isoforms (GluN2A-GluN2D) present in the brain [33]. Each subunit contains four membrane-bound domains, three of which are transmembrane (M1, M3, and M4), and one (M2) is a re-entrant pore loop [34].

The modulation of NMDA receptors occurs via multiple mechanisms [35], although ethanol functions directly as an allosteric NMDA receptor inhibitor, reducing the frequency and duration of channel opening. This occurs by interacting with specific modulatory sites within the receptor. These include a limited number of positions in the GluN1 subunit of the M3 domain and in the GluN2A subunits of the M3 and M4 domains: Phe-639 in GluN1 M3, the related Phe-637 in GluN2A M3, and the Met-823 and Ala-825 positions in GluN2A M4 [36]. The mechanism of action of ethanol on the positions of Phe-637 in GluN2A and Phe-639 in GluN1 involves modification of the hydrophobic region by stabilizing the non-conductive state of the channel, which undergoes closure or desensitization [34,37]. In the case of the M4 domain and the positions of Met-823 and Ala-825, ethanol exhibits an indirect effect by modulating interactions within the M4 domain. This modulation involves the disruption of dynamic relationships between transmembrane residues and interactions with the M3 domain itself. This, in turn, affects changes in gating rate and the stabilization of the desensitized state [36]. Consequently, this reduces ionic conductance within the channel [38].

### 2.3. The Dopaminergic System

Ethanol consumption has been demonstrated to induce the release of dopamine (DA) and endogenous opioids, also known as peptide neurotransmitters, in the ventral striatum [39]. Dopamine, a key catecholamine, is produced in the black matter (SN) and VTA of the midbrain [40]. The consumption of addictive substances, such as alcohol, has been demonstrated to enhance dopaminergic system stimulation [39]. This phenomenon is attributed to an escalation in the spontaneous discharge rate of dopaminergic neurons within the VTA, which is contingent upon the ethanol dosage [41]. The mesolimbic DA circuit is constituted by dopaminergic neurons in the VTA and their projections to several brain areas, primarily the nucleus accumbens (NAc). The term “group A cells” is used to denote groups of DA-producing cells, while those cells that also produce catecholamines, chiefly norepinephrine and DA, are classified as group A8 to A16 cells. The A10 cells, in particular, are located in the VTA and project to the NAc, prefrontal cortex (PFC), and other limbic areas. These cells constitute the mesolimbic and mesocortical pathway, which is responsible for positive and negative reward-related reinforcement and motivational conditioning [40], as evidenced by their role in alcohol use [39].

The prolonged exposure to addictive substances results in neuroadaptation of the circuitry of the basal nuclei, a pivotal mechanism in the subsequent development of compulsive drug-seeking habits. Chronic alcohol consumption can also lead to allosteric changes that are necessary for normal brain function [39,42]. The most severe form of Alcohol Withdrawal Syndrome (AWS) is Delirium tremens (DT), which occurs in chronic heavy drinkers who experience a sudden reduction or complete cessation of alcohol consumption. The symptoms associated with DT include disorientation, confusion, hallucinations, tremors, and autonomic agitation and instability [43]. An illustration of the aforementioned allosteric change is the dysfunction of dopaminergic transmission of dopamine in nigrostriatal and mesolimbic pathways [39,42]. This phenomenon can be attributed to a decrease in the number of D2 receptors and dopaminergic activity. This, in turn, reduces the sensitivity of reward circuits to stimulation by natural reinforcers, leading to decreased basal dopamine [44]. This decrease in dopamine can occur as a result of alcohol withdrawal after a prolonged episode of alcohol consumption. Concurrently, the development of behaviors indicative of alcohol dependence ensues. The hypodopaminergic state can serve as a catalyst for the emergence of alcohol craving, manifesting as alcohol seeking and subsequent use [45,46]. Specifically, low concentrations of DA can be detected in the caudate nucleus of the brains of alcoholics [46].

### 2.4. Stimulation of the Opioid System

The opioid system occupies a pivotal position in the process of nociception and the management of pain. Beyond this, it has been demonstrated to regulate a multitude of physiological functions, including stress responses, respiration, and endocrine and immune functions. It also plays a fundamental role in modulating mood and well-being and addictive behavior by regulating the rewarding properties of drugs such as alcohol [44].

The classical endogenous opioid system comprises three types of G-protein-coupled receptors: μ receptors (MOR), δ receptors (DOR), and κ receptors (KOR). Their specific ligands include endorphins, enkephalins, and dynorphins. The sources of endogenous opioid peptides are peptide precursors, or prohormones. Beta-endorphin, which exhibits a high affinity for μ-opioid receptors, is synthesized through the process of proteolytic conversion of proopiomelanocortin (POMC). Dynorphin B, a selective activator of κ-opioid receptors, is a byproduct of prodynorphin. Conversely, Met-enkephalin-Arg6-Phe7 (MEAP), derived from proenkephalin, functions as an agonist at the δ-opioid receptor, while exhibiting some degree of affinity for μ-opioid receptors [47].

Valenza et al. present observations on the rewarding effect of alcohol, which is mediated by the opioid receptor system through the release of endogenous opioids, including beta-endorphins. These opioids are then bound by opioid receptors in the mesocorticolimbic system. Of particular relevance to the positive effects of alcohol reinforcement are the μ and δ subtypes. The μ opioid receptor, upon activation, has been shown to remove the inhibitory tone of GABAergic interneurons in the VTA, leading to increased dopamine release. Concurrently, the activated μ opioid receptor present in the shell of the NAc has the capacity to enhance the motivational properties of alcohol [48].

These enhanced dopamine levels in the ventral striatum activate D1-MSNs, which contribute to various adaptations, such as changes in dynorphin regulation resulting from activation of the transcription factor CREB in NAc neurons. Increased dynorphin expression is a factor in the inhibition of dopamine release through activation of KOR. These receptors are present on the presynaptic terminals of dopaminergic neurons in target structures and on the dopaminergic cell bodies of the VTA. A reduction in overall dopaminergic transmission has been shown to shift the point of dopamine homeostasis, a direct factor in the development of hypercathepsy, characterized by an elevated sensitivity to aversive stimuli, including pain and negative emotional experiences. Furthermore, a reduction in dopaminergic activity contributes to anhedonia, defined as an impaired ability to experience pleasure [49].

Ethanol consumption has been demonstrated to stimulate the expression of enkephalins (ENKs) in the paraventricular nucleus of the hypothalamus (PVN) and in numerous extra-thalamic mesocorticolimbic nuclei, including the VTA, the NAc, the medial prefrontal cortex (mPFC), and the central nucleus of the amygdala (CeA) [50]. It is noteworthy that enkephalins, the endogenous ligands of MOP and DOP receptors, exhibit a more than 10-fold higher affinity for the DOP receptor [51].

Beta-endorphins, similar to enkephalins, are also ligands for MOP and DOP receptors, with comparable binding affinities [50]. These neurotransmitters are linked to the hypothalamic–pituitary–adrenal (HPA) axis, which plays a crucial role in regulating various physiological functions, including the body’s response to stress and the production of analgesic effects [52].

## 3. Medicinal Products in the Treatment of Alcoholism

The roots, symptoms, and treatment of AUD can vary between patients. Not everyone who drinks alcohol develops AUD, and even those who develop it do not usually meet all 11 criteria of AUD, as only meeting two of the eleven is enough to diagnose AUD [53].

The interpatient clinical picture is driving the development of increasingly sophisticated treatment strategies for AUD. Treatment of AUD consists of using pharmacological, non-pharmacological options or a combination of these two [39].

Non-pharmacological treatment of AUD should be advised based on patients’ needs. Brief motivational interventions are performed by physicians or health care professionals, and the goal is to encourage patients to improve their lifestyle. The patient should be provided with support and feedback [54]. This type of therapy can be unsuccessful in the long term, and it depends on the length of therapy. For patients with severe AUD, inpatient residential treatment should be taken into consideration [39].

Currently, there are only a few medications approved by the U.S. Food and Drug Administration (FDA) to treat AUD. These drugs include disulfiram, acamprosate, and naltrexone [1]. Nalmefene, a drug used in the treatment of AUD in Europe, has not been approved by the FDA yet [53].

Although the relative clinical effectiveness of nalmefene and naltrexone in the treatment of AUD remains a subject of ongoing debate, comparative evidence on the superiority of either compound has yet to yield definitive conclusions. This review presents a detailed analysis of the pharmacological, pharmacodynamic, and pharmacokinetic characteristics of these two compounds and compares their clinical profiles. A thorough understanding of the pharmacological properties and differences between these two compounds may facilitate the development of more individualized therapeutic approaches.

### 3.1. Naltrexone

#### 3.1.1. Pharmacological Characteristics

Naltrexone has received the most extensive research attention of the three currently approved drugs in the context of treating AUD in adults, as approved by the Food and Drug Administration [55,56]. Originally marketed for the treatment of opioid use disorder, it remains the most widely used due to its antagonistic effects on opioid receptors [56]. The medication is available in oral and injectable forms, with the injectable formulation exhibiting a longer duration of action [57].

The Anatomical Therapeutic Chemical (ATC) classification code for Naltrexone is: N07BB04. It is classified within the group N07B, which encompasses drugs for addictive disorders, and more specifically, within the following subgroups: N07BB, which includes drugs for alcohol addiction, and N07BC, which includes drugs for opioid addiction [58].

#### 3.1.2. Pharmacodynamic Properties

Naltrexone, formulated as naltrexone hydrochloride, is a μ opioid receptor antagonist that also exhibits affinity for κ and δ opioid receptors [56,59]. It has been demonstrated that naltrexone exerts its effects by competitively binding to receptors located within the central and peripheral nervous systems, thereby impeding the actions of endorphins and opioids, including β-endorphins, which have been implicated in the experience of euphoria in individuals diagnosed with AUD. The effects of naltrexone include prolonged abstinence time, overall reduced alcohol consumption, and a reduction in the percentage of adults relapsing after long episodes of drinking [11,55]. Laboratory studies have also demonstrated a reduction in the hedonic pleasure associated with alcohol, particularly among heavy and problem drinkers [55].

#### 3.1.3. Pharmacokinetic Properties

Following oral administration, naltrexone hydrochloride rapidly enters the gastrointestinal tract. The maximum plasma concentration of the drug is reached after the first hour, with a plasma protein binding of 21% and a steady-state concentration of 8.55 mg/mL [59]. The primary metabolic pathway of naltrexone hydrochloride occurs in the liver via a first-pass mechanism [59,60]. The enzymes responsible for the conversion of naltrexone hydrochloride are members of the aldo-keto reductase 1C (AKR1C) family, specifically AKR1C4, with a lesser contribution from AKR1C1 and AKR1C2 [61]. The resultant hydroxylation reaction yields the active metabolite 6-beta-naltrexol, whose plasma concentrations are elevated to levels 10 to 40 times those of naltrexone alone, with 2-hydroxy-3-methoxy-6-beta-naltrexol being a less significant metabolite [59,60,61].

The drug’s primary route of elimination is the kidneys. Within the first 48 h of administration, approximately 60% of the oral dose is excreted as 6-beta-naltrexol glucuronate and naltrexone hydrochloride [59]. Naltrexone has a half-life of 3.9–10.3 h, with an extended elimination half-life of 96 h. In contrast, the serum half-life of 6-beta-naltrexol is longer at 13 h [59,60].

Intramuscular administration of naltrexone has been demonstrated to circumvent the first-pass effect. Consequently, there is a shift in the exposure ratio of 6-β-naltrexol to naltrexone in comparison to oral administration. The concentrations of 6-β-naltrexol reach values that are only 4 times higher than those of naltrexone. Consequently, the average half-life of 6-β-naltrexol is observed to be approximately five to ten days, which is equivalent to that of the parent compound [61,62].

### 3.2. Nalmefene

#### 3.2.1. Pharmacological Characteristics

Nalmefene is a pharmaceutical agent employed in the treatment of AUD. The administration of this pharmaceutical agent can be performed via intravenous (IV), intramuscular (IM), subcutaneous (SC), or intranasal (IN) routes [56,62,63]. ATC classification code for Nalmefene is: N07BB05. It is also found in group N07B, drugs for addictive disorders, and subgroups: N07BB, or drugs for alcohol addiction, and N07BC, or drugs for opioid addiction [58].

#### 3.2.2. Pharmacodynamic Properties

Nalmefene exhibits structural similarity to naltrexone, with the key difference in its chemical structure being the presence of a 6-methylene group in place of the 6-ketone group characteristic of naltrexone [63]. This subtle structural modification results in comparable functional properties for both compounds. Nalmefene, akin to naltrexone, functions as an antagonist of opioid mu and delta receptors, thereby delineating its mechanism of action [56,63]. The presence of a methylene group in Nalmefene contributes to an augmented duration of action (DOA) and an elevated affinity for the MOR [63]. Nalmefene also functions as an agonist of the KOR, which is predominantly located in the dopaminergic circuits of the NAc. Its actions can result in a reduction in the motivation to consume alcohol, both spontaneously and in the context of withdrawal [56]. This phenomenon can be attributed to the inhibition of positive reinforcement within the mesolimbic reward system pathway [63].

Nalmefene, when administered orally, has been shown to exert its effects on MOR and KOR. These effects include the inhibition of the release of β-endorphins, which are MOR agonists, and dynorphins, which are KOR agonists. This inhibition of opioid receptor activity has been demonstrated to result in a reduction in alcohol consumption [63].

#### 3.2.3. Pharmacokinetic Properties

The oral administration of Nalmefene results in rapid absorption, with maximum concentrations achieved approximately 1.5 h after administration. The extent of drug binding to proteins is approximately 30%. Furthermore, Nalmefene exhibits a notable capacity to traverse the blood–brain barrier, suggesting its potential for systemic action. This is evidenced by the occupation of 94% to 100% of opioid receptors within 3 h of drug administration. Following oral administration, the primary metabolic pathway of Nalmefene leads to the formation of Nalmefene 3-O-glucuronide, accompanied by the less significant production of Nalmefene 3-O-sulfate, a byproduct resulting from reactions with sulfuric acid and Nornalmefene. Subsequent to its initial conversion, Normalmefene is subject to further transformation, resulting in the formation of two distinct derivatives: nornalmefene 3-O-glucuronide and nornalmefene 3-O-sulfate. The majority of these products demonstrate minimal interaction with opioid receptors; however, Nalmefene 3-O-sulfate is an exception, exhibiting a potency comparable to Nalmefene. The primary route of elimination of Nalmefene from the body is via glucuronic acid conjugation in the liver. Consequently, both the drug itself and its metabolites are transported to the kidneys, where they are excreted. The most significant metabolite is Nalmefene 3-O-glucuronide, which accounts for 54% of the total original dose of the drug. The presence of other metabolites and Nalmefene in the blood is negligible, with concentrations below 3% each recorded. The oral form of the drug has a half-life of 12.5 h, while its intravenous administration yields a comparable half-life of 10.8 h. Notably, there is a lack of substantial variance in pharmacokinetics across gender, age, or ethnic groups. Conversely, the clearance of Nalmefene is reduced by approximately 28% in patients with mild to moderate liver dysfunction [62]. A comparison of the two drug products is provided in Table 1.

## 4. Comparison of the Clinical Profiles of Naltrexone and Nalmefene

### 4.1. Objective of the Clinical Comparison

The potential for modulating the opioid system to treat alcoholism and reduce alcohol consumption is enabled by the availability of two medications: naltrexone and nalmefene. Given the differential effects on different types of opioid receptors, it is reasonable to expect different therapeutic effects [56].

The purpose of the review is to contrast Naltrexone and Nalmefene in the context of the most relevant parameters in the treatment of alcoholism: total alcohol consumption, daily alcohol consumption, number of drinking days per month, number of heavy drinking days per month, and possible reduction in the hepatotoxic effects of alcohol. Hepatotoxicity will be assessed by liver tests, among others. Analysis of large data conducted on specific subpopulations may prove crucial in planning personalized pharmacotherapy for alcoholism. The individualization of pharmacotherapy also requires, among other things, an awareness of the safety profile of both drugs, possible routes of administration, and treatment regimens. This review is intended to address all of the above factors.

### 4.2. Original Studies on Nalmefene

The EMA’s approval of Nalmefene was preceded by studies evaluating its clinical efficacy and safety profile. These were Phase III, randomized, double-blind, placebo-controlled clinical trials: ESENSE1, ESENSE2, and SENSE, the first two of which included 6 months of primary treatment in patients with a WHO Drinking Risk Level (DRL) of moderate to very high, and the last of which included 13 months of treatment in patients with a DRL of low to very high. Each study used Nalmefene 18 mg, taken orally, in adults on an as-needed regimen [64].

In parallel with treatment, in each of the 3 studies, participants were supported by the BRENDA program aimed at maintaining the subjects’ motivation, with the ultimate goal of improving their compliance with treatment recommendations [65,66,67].

The Brenda model was created to maximize the patients’ compliance with doctors’ orders, including taking prescribed medication, by using a six-step approach. The first step is to evaluate the patient, both physically and mentally. Then, all information obtained should be reported back to the patient. The next two steps are empathy and addressing the patient’s needs. The fifth step is to give the patient advice on how to deal with the problem. Finally, the doctor should consider patient reaction and if necessary, modify treatment plan [68].

The ESENSE1 results and study were published by Mann et al. [65] It included 604 subjects who, in addition to those mentioned above, met the criteria of at least six days of heavy drinking per month (for men, one day of heavy drinking is defined as >60 g of ethanol consumed, for women >40 g of ethanol consumed) over a four-week period prior to screening. Finally, 289 patients in the placebo group were included in the efficacy comparison, along with 290 patients who took Nalmefene 18 mg orally as needed—it was recommended that the drug be taken several hours before the expected episode of alcohol consumption. After 24 weeks, the efficacy of Nalmefene was compared to placebo: HDD (heavy drinking days per month) lower by 2.3 days (95% CI −3.8; −0.8; *p* = 0.0021), TAC (total alcohol consumption per day) lower by 11 g/day (95% CI −16.8; −5.1; *p* = 0.0003). A statistically significant difference in both parameters in favor of Nalmefene was observed in the first month and was maintained throughout the treatment period. In contrast, the overall incidence of treatment-emergent adverse events (TEAEs) was 14.6 percentage points higher in the Nalmefene group. The rate of TEAEs leading to discontinuation was 22.8% in the study group and 7.4% in the control group. Improvements in liver enzymes at the end of the study were greater in the Nalmefene group for both γ-glutamyltransferase (GGT) and alanine aminotransferase (ALAT), but statistically significant only for GGT (GGT: *p* = 0.0094; ALAT: *p* = 0.0109) [65].

A total of 718 patients were enrolled in the ESENSE2 twin study published by Gual et al. [67] 201 patients in the Nalmefene group and 210 in the placebo group completed the main treatment. The methodology and exclusion criteria were the same as in ESENSE1. After 24 weeks of the study, HDD was reduced by 1.7 days compared to placebo, and TAC was decreased by 5 g/day compared to placebo. A statistically significant advantage for Nalmefene was observed for both values after the first month. In addition, the effect of Nalmefene was even stronger in patients who did not independently reduce their alcohol intake between screening and randomization: HDD-2.0 and TAC-7.0 compared to placebo at the end of week 24. The incidence of adverse events was 9.0 percentage points higher in the study group than in the control group, with the percentage of TEAEs leading to discontinuation being 6.7% and 5.9%, respectively. GGT and ALAT profiles improved more in the Nalmefene group, but a statistically significant difference was observed only for ALAT (GGT: *p* = 0.529; ALAT: *p* = 0.049) [67].

The last of the studies prior to the European Medicines Agency (EMA) decision was SENSE, designed by van den Brink et al. [66]. 675 patients with AUD were randomized in a 1:3 placebo/nalmefene ratio. 112 and 310 patients, respectively, completed the main treatment. Despite a wider overall range of DRLs among subjects (low to very high) than in ESENSE1 and ESENSE2, in SENSE the authors define the study’s target population as patients with high/very high DRL. At month 13, statistically significant differences in alcohol consumption parameters in favor of the drug were observed. HDD was reduced by 1.6 days/month and TAC by 6.5 g/day. There was also an advantage of the drug over the placebo for the tabulated values of both liver tests. Corresponding data for the target population (DRL high/very high) are also presented. In these subjects, the decline in drinking parameters at month 13 was more than double that of the overall study population: HDD decreased by 3.6 days/month and TAC by 17.3 g/day. The incidence of all adverse events was 12 percentage points higher in the nalmefene group than in the placebo group. In addition, 11.4% of all TEAEs in patients taking nalmefene led to discontinuation, compared to 3% in the placebo group [66].

Regulatory approval to manufacture and sell the first nalmefene hydrochloride formulation in Japan was granted to Otsuka Pharmaceutical Co., Ltd. (OPC) on 8 January 2019 [69]. Previously, OPC conducted 3 studies to demonstrate the drug’s efficacy, safety, tolerability, and pharmacokinetics in Japanese individuals aged 20 and older. The first was small (n = 7) and evaluated only the pharmacokinetics and tolerability of a single dose of the compound. The second was already a Phase III, multicenter, randomized, placebo-controlled clinical trial. It was primarily designed to evaluate the effect of nalmefene on reducing alcohol consumption in patients with AUD. It represents a lead-in study, as the third study was an extension of the second study and was conducted to determine the long-term efficacy and safety of the drug [70,71,72].

A summary of the results of the lead-in study was published by Miyata et al. [64] The study enrolled 677 subjects who had to meet the criteria of high/very high DRL. They were randomized to the following groups: nalmefene 20 mg, nalmefene 10 mg, and placebo. The treatment was completed by 189, 139, and 219 subjects in the three groups, respectively. They were to take the assigned formulation as needed for 24 weeks. In addition to the pharmacotherapy itself, all patients simultaneously participated in a support program based on the BRENDA model used in the European ESENSE and SENSE studies. The study results at week 12 and week 24 showed a statistically significant difference in favor of nalmefene (both doses) in both HDD and TAC. At month 6, nalmefene 20 mg reduced HDD by 3.92 days/month [95% CI −5.69; −2.16; *p* < 0.0001] and TAC by 11.15 g/day [95% CI −16.77; −5.53] compared to placebo. Overall, patients receiving nalmefene experienced 7.4 percentage points more adverse events per capita than patients receiving a placebo. In addition, the TEAEs, which are more likely to lead to discontinuation, occurred less frequently in the nalmefene 10 mg group than in the nalmefene 20 mg group. The nausea was reported by 6.5% of patients treated with nalmefene 20 mg and by 8.5% of patients treated with nalmefene 10 mg. In case of dizziness, it was 4.9% compared to 6.5%; and in case of vomiting, 4.0% compared to 2.7%. Improvements in liver enzyme profile were statistically significantly greater for nalmefene 20 mg than placebo at both weeks 12 and 24 for GGT and ALAT, and there was also greater improvement in the nalmefene 10 mg group, although ALAT was not statistically significant at any time point in the study.

Higuchi et al. [69] published an extension study based on a cohort of patients who completed the initial study described above and agreed to continue participation. The follow-up consisted of a 24-week, open-label, non-controlled study based on the use of nalmefene 20 mg by all patients regardless of the substance taken before. A total of 343 subjects completed this part of the study and were then randomized to receive nalmefene 20 mg or placebo in a 1:1 ratio for a 4-week double-blind, placebo-controlled run-out period, followed by a 4-week follow-up period during which subjects did not take the drug. Over the course of the 24 weeks of the main study, there was a decrease in the total number of TEAEs reported by patients taking the drug at the 20 mg dose in both the lead-in and extension studies. There was also a significant reduction in the incidence of the most commonly reported events in the lead-in study, including nasopharyngitis (24.1% of patients receiving placebo/16.8% of patients treated with nalmefene), nausea (27.7%/15.3%), and excessive somnolence (19.0%/6.6%), while the proportion of serious TEAEs and those leading to discontinuation did not change significantly. During the run-in period, 17.4% and 11.7% of participants reported adverse events per person for nalmefene and placebo, respectively, including one serious adverse event in each group.

In contrast, no serious TEAEs were reported during the final 4-week follow-up period. Adherence was also determined to be 80.2% for subjects who experienced a TEAE and 86.2% for the minority of subjects who did not experience a TEAE. The study was also designed to assess the risk of nalmefene dependence symptoms during the run-in period, when one group was taking nalmefene and the other a placebo, and to assess possible withdrawal symptoms during the follow-up period, when neither group was taking the drug. The assessment was based on questionnaires in which subjects rated the severity of the reported symptom. The results of the Dependence Assessment Questionnaire showed that every single symptom suggested by the subjects was more frequent in the placebo group than in the nalmefene 20 mg group, indicating that the drug had no addictive potential. In contrast, in the withdrawal assessment, only 2 patients in the placebo group reported the presence of withdrawal symptoms, while none of the patients in the nalmefene group reported the presence of withdrawal symptoms [69].

The results of the extension study also demonstrated the long-term efficacy of nalmefene in the study population. In the group of patients taking nalmefene 20 mg, reductions in drinking severity parameters were observed over 48 weeks of treatment. HDD was 13.8 ± 0.76 days/month lower than baseline at week 28, i.e., one month after the start of the extension study, and 15.09 ± 0.77 days/month lower at week 48. The TAC values were −48.45 ± 2.35 g/day and −53.20 ± 2.29 g/day, respectively. This indicates maintenance of the drug’s therapeutic effect and further reductions in drinking parameters for at least six months after the initial rapid clinical effect [69].

Another study based on a large cohort of patients, conducted to evaluate the efficacy and safety of nalmefene, is a 12-week interventional, open-label study published by Castera et al. [73]. Only participants with a high DRL were enrolled in the screening study, after which, after 2 weeks, they were enrolled in their respective groups of the relevant part of the study according to their alcohol consumption during the previous 2 weeks—participants who maintained a high DRL in cohort A (n = 330) and those who reduced their DRL below high in cohort B (n = 48). Those in cohort A received nalmefene 18 mg for 12 weeks in an as needed + psychosocial support regimen, while those in cohort B received only the latter intervention. The results in terms of differences in HDD and TAC between the screening visit and the end of the open-label study in the drug-treated group were as follows HDD reduction of 13.1 days/month (95% CI −14.4; −11.9), TAC was reduced by 64 g/day (95% CI −69.4; −58.6). The results indicate significant short-term efficacy of nalmefene, comparable to the previously cited RCTs. Improvements in liver enzymes were also observed, with both GGT and ALT levels improving significantly over the course of treatment. Adverse events occurred in 64.0% of participants in cohort A, the most common being nausea, dizziness and insomnia. Only 6.4% of patients withdrew from the study due to more severe TEAEs [73].

In the last decade, 4 studies have also been published that evaluated the effect of nalmefene on the severity of AUD but based on a smaller and/or narrower population in terms of inclusion criteria. Table 2 presents the data from these studies or the conclusions drawn from them.

### 4.3. Original Studies on Naltrexone

Naltrexone, as a form of targeted AUD treatment, was approved by the FDA as early as 1994 and is thus clearly better studied and described than nalmefene, which is described above. In this case, a much larger number of meta-analyses, reviews, and thus original clinical studies in different populations are available. In 2006, the FDA approved Naltrexone as a long-acting injection (LAI), further expanding the target population and enabling its efficacy to be studied in a larger proportion of the target population [78,79].

LAI consists of monthly intramuscular injections of an extended-release medication, accompanied by behavioral psychotherapy. A systematic review and meta-analysis of studies on this form of treatment were published by Murphy et al. [78]. It focused on seven studies with a control trial, in which the doses of naltrexone ranged from 150 to 400 mg, and the duration of treatment ranged from 2 to 6 months (and therefore from 2 to 6 injections taken by the patient during the entire treatment). They included a total of 1500 patients with different characteristics, but all with AUD of moderate to severe according to DSM-IV. The authors analyzed the following parameters, among others: number of drinking days per month (DD) and number of heavy drinking days per month (HDD). Due to the large number of studies, the study samples were compared with the control samples using a pooled weighted mean difference (WMD), which allowed the previously assessed precision of the individual studies to be included in the comparison. For both DD and HDD, the difference was statistically significant—WMD for DD: −2.0 [95% CI −3.4; −0.6; *p* = 0.03] in favor of naltrexone over placebo, WMD for HDD: −1.2 [95% CI −0.2; −2.1; *p* = 0.02] in favor of naltrexone. Four studies were also used to assess the risk of relapse to heavy drinking. Still, the results indicated no statistically significant difference between the drug and placebo groups [78].

A recent meta-analysis by McPheeters et al. [79] summarized most of the medications used in the pharmacotherapy of AUD in the form of results from 12-week RCTs in subjects with mostly moderate to severe AUD. Three additional RCTs on the clinical efficacy of the LAI form are included in the publication. The results for baseline drinking parameters were as follows—WMD for DD: −4.99 [95% CI −9.49; 0.49], for HDD: −4.68 (95% CI −8.63; −0.73). At the same time, the mean results for the parameters are presented for studies on other naltrexone dosage forms and dosing regimens. The 15 studies where naltrexone was taken PO at a dose of 50 mg per day showed WMD for DD: −5.1 (95% CI = −7.16; −3.04), for the same dose, the difference in HDD was studied by 7 studies—WMD: −4.3 (95% CI −7.6; −0.91). The study of a higher dose (100 mg/d) of naltrexone is much less common. In this case, only 3 studies presented their efficacy in terms of DD and 2 in terms of HDD. The results were—WMD: −2.3 [95% CI = −5.6; 0.99] and −3.1 [95% CI −5.8; −0.3], respectively. Thus, there is a difference in parameter reductions across the drug’s dosing regimens, although the difference in the number of studies between the doses is too large to evaluate one regimen versus another. However, it must be said that naltrexone taken orally at a dose of 50 mg/d is the best described and studied form of pharmacotherapy for alcoholism using substances active against the opioid system, and its effectiveness is clearly confirmed, among others, by a meta-analysis by McPheeters et al. Despite the existing indications for the LAI form in specific cases, the safer first-line drug will therefore be the oral form at a dose of 50 mg/d [64,79].

Among cases indicating a higher likelihood of clinical success with an injectable form of naltrexone, the most common is associated with potential patient adherence issues. Lower adherence is significantly associated with younger age, homelessness, and use of other psychoactive substances, particularly cocaine. Closely focused on the issue of pharmacotherapeutic adherence in the treatment of AUD with naltrexone is a study by Bernstein et al. [80]. In the course of the study, 244 people were randomly assigned to two groups: 124 to the PO drug group, 120 to the LAI group. After 12 weeks of treatment, adherence was determined for each group—for PO based on a post-treatment questionnaire, and for LAI on the basis of the number of injections of the drug received in the hospital (treatment assumes an injection at a medical facility at a monthly follow-up visit). In order to standardize the measurement, it was assumed that the minimum indicative of adherence to treatment was 60 days of oral intake or receipt of at least 2 monthly injections. The results between groups differed significantly: 37.9% adherence for the PO group, 62.5% for the LAI group. It suggests a much higher potential for the injectable form over the oral form for patients in groups with statistically lower adherence, as mentioned above and detailed in Bernstein’s analysis. Also not without influence in cases of potentially reduced adherence are possible interventions aimed at controlling adherence and/or improving other living conditions, including those related to risk factors for reduced adherence [80].

An example of such an intervention is the Medication Event Monitoring System (MEMS), which was the basis of a study conducted by Stoner et al. [81] and secondarily analyzed over time by Dermody et al. [82] The main objective of the secondary analysis was to detect and identify daily factors predictive of medication adherence based on patient reports and responses to the developed system. The author reports that nowadays, due to the greater availability of reports on the impact of patients’ baseline characteristics on this phenomenon, factors of daily life provide more room for consideration and research. Data from 56 participants were used for secondary analysis. Measurement procedures and measures are described in the publication. Among other things, they found a general decrease in adherence over time, particularly on days following days of increased drinking, and an association of increased adherence with reliable completion of the daily mobile self-assessments that are part of the MEMS. Dermody et al. [82] aptly point out the need to work on a strategy to effectively return to prescribed pharmacotherapy after episodes of heavier drinking or broken abstinence. At the same time, it describes the disadvantage of mobile control systems, namely their inapplicability to a significant proportion of affected AUDs, and reminds us of the LAI form of naltrexone, which, according to the cited sources, would be more effective for the target groups addressed [83,84].

Extended-release naltrexone (XR-NTX) was also used in a randomized clinical trial involving one of the key subpopulations associated with reduced adherence—homeless people. Its analysis was published by Collins et al. [85]. Participants included 308 people exhibiting 67.0% severe AUD, who were randomized to the following 4 arms: behavioral Harm-Reduction Treatment for Alcohol (HaRT-A) only, HaRT-A + LAI naltrexone, HaRT-A + injections of placebo, or treatment as usual (TAU) only. They lasted 12 weeks, but only 52% of the TAU group showed up for evaluation at week 12, when the average was 70% in the HaRT-A groups. This indicates a favorable effect of the more involved and individualized care (which, unlike TAU, characterized HaRT-A) on patients’ propensity to continue treatment (An explanation of the pattern of the various interventions and a description of the measures taken is provided in the source publication). In contrast, no significant differences in the frequency of adverse events were observed between the different arms. To approximate the effect of the drug itself on drinking parameters, compare the HaRT-A + LAI naltrexone group with the HaRT-A + placebo injections group. According to the week 12 evaluation, the parameter for standard drinks on peak occasion for the drug group was 12.81 (SD = 3.30), and for placebo, 19.09 (2.28). On the other hand, DD averaged 15.15(11.33) for NTX and 18.68(11.45) for placebo [85].

A different analysis of the aforementioned study, published by Vutien et al. [86] ruled out an association of extended-release naltrexone with hepatotoxicity in homeless individuals experiencing active AUD (96.7% physiologically dependent). The study evaluated the serum levels of three liver enzymes: aspartate aminotransferase (AST, AspAT), alanine aminotransferase (ALT, ALAT, AlAT), and GGT. Each result was classified into the appropriate group: normal level (<1x upper-limit-of-normal [ULN]), elevated level (1-3xULN), or high level (>3xULN). At week 12 for the NTX group, 60.4% of subjects were classified with normal AST, 32.1% with elevated AST, and 7.6% with high AST. ALAT levels were observed, respectively: 84.9%/11.3%/3.8%, and GGT: 62.3%/26.4%/11.3%. In the placebo group AST: 45.8%/41.7%/12.5%, ALAT: 70.8%/25.0%/4.2%, GGT: 47.9%, 29.2%, 22.9%. Furthermore, at no stage of treatment was the difference in the range of serum enzyme increases between the XR-NTX group and the placebo group statistically significant. The findings suggest that the administration of naltrexone LAI is generally safe for patients with AUD, including those with severe addiction and indications of physiological dependence [86].

A more problematic subpopulation in the context of AUD treatment is adolescents, due to differences in the structure of the central nervous system and its ongoing development, including brain regions that determine sensitivity and susceptibility to alcohol, which may have important implications in the way compounds active against opioid receptors affect teenage drinking. An RCT involving such a subpopulation was conducted by Robert Miranda et al. The 22 adolescents aged 15–19 years exhibiting an AUD took naltrexone 50 mg PO or placebo for 8–10 days. The results showed a statistically significant lower propensity to drink alcohol at all by 31% (*p* = 0.03) in the naltrexone group relative to placebo, and a lower risk of heavy drinking by 54% (*p* = 0.003). These provide experimental evidence of the drug’s potential for use in the context of the AUD-affected subpopulation studied as well, and point to the need for further studies with similar methodology [83].

The key clinical studies evaluating naltrexone in the treatment of AUD are summarized in Table 3.

## 5. Discussion

AUD is a serious social problem with significant consequences, as evidenced by health statistics. The data presented by the WHO reveals alarming statistics regarding the number of deaths occurring per year and the significant social costs associated with this phenomenon. AUD is characterized as a neurobiological and psychosocial dysfunction, which has been demonstrated to exert an influence on the elevated suicide rate. It is imperative to acknowledge that this condition represents the second most significant risk factor for suicide, subsequent to affective disorders.

It is important to note that the long-term consumption of alcohol can lead to abnormalities in cognitive functions, excessive impulsivity, and long-term changes in brain function, particularly in memory and cognitive abilities. The neurodegenerative effects associated with alcoholism are primarily due to its effects on the GABAergic and glutamatergic systems—it impairs GABAergic activity and increases the sensitivity of NMDA receptors. This results in significant consequences for cognitive processes through neuromodulatory mechanisms. The effects of ethanol on the functioning of these systems have been repeatedly studied in animal models, especially in laboratory mice. The effects of ethanol on the glutamatergic system determine the mechanisms of neurotoxicity and neuroadaptation. Ethanol is an allosteric inhibitor of NMDA receptors, and its long-term effects lead to the increased sensitivity of these receptors, which are responsible for the pathomechanism of convulsions and delirium tremens. Chronic alcohol abuse also affects the dopaminergic system, which plays a key role in motivational, emotional and reward-related processes. An often-overlooked effect of alcohol consumption is the release of β-endorphins, enkephalins, and dynorphins, which stimulate opioid receptors.

Pharmacological solutions for treating alcoholism have been developed that are based on antagonism to these receptors. These solutions result in reduced satisfaction with alcohol, consequently reducing addiction. The preferred pharmaceutical interventions for this purpose may include Naltrexone and Nalmefene. Analysis of available review papers and clinical trials [75,76,77,86] confirms the clinical efficacy and lack of contraindications in the safety profile, including hepatic safety, for the use of both described drugs. Reduction in the severity of drinking, measured most often through parameters: HDD, DD, TAC in the pharmacotherapy groups was statistically significant (based on the difference relative to placebo, in favor of the drug) in the trials analyzed [78,85,86]. Differences in trial methodology-characteristics of study groups, dosing regimen, method of measurement, and lack of head-to-head studies preclude direct comparison of the effectiveness of the two drugs. Indeed, the larger number of studies on naltrexone influences its superiority over nalmefene [84,87].

In addition to the more numerous studies based on a large cohort [78,79,80], those with narrow, strict inclusion criteria have also been conducted to examine the drug’s effect on specific subpopulations—e.g., adolescents [83], homeless people [85,86]. The wider range of naltrexone formulations available for treatment and the presence of studies confirming their efficacy and hepatic safety make the target group of AUD sufferers broader for naltrexone than for nalmefene [86].

The (LAI formulation) is well-suited for people who show reduced adherence to previous treatment recommendations, including those related to pharmacotherapy. It significantly reduces the effort required of the patient to continue pharmacotherapy, increasing the likelihood of clinical success. Nalmefene does not have a registered LAI formulation or any injectable formulations for the treatment of AUD. However, Nalmefene has been extensively studied under an as-needed dosing regimen (ESENSE1, ESENSE2, SENSE studies), and these studies confirm its effect on reducing alcohol consumption when taken on this basis. The ability to use it in this way increases the convenience of pharmacotherapy, which may encourage those showing a desire to reduce their drinking to implement it.

## 6. Conclusions

The issue of AUD has gained global prominence. An increasing number of individuals are consuming alcohol on an annual basis, which is concomitant with a rise in the prevalence of AUD. This problem is the foundation for the development of the most effective treatment for AUD. The non-pharmacological treatment can be unsuccessful in the long term; therefore, pharmacological options should be considered, with medication that affects opioid receptors, such as naltrexone and nalmefene, being particularly promising.

Naltrexone is typically administered daily as part of a treatment regimen, while nalmefene is reserved for emergencies. The administration of both medications yields the anticipated outcomes. A decline in the prevalence of heavy drinking days has been documented. It is evident that both medications are well tolerated and possess a satisfactory safety profile. Naltrexone has also been the subject of clinical investigation in groups of homeless patients and adolescents, with a view to demonstrating its effectiveness in these particular demographics.

A direct comparison of naltrexone and nalmefene is only possible if head-to-head studies are conducted, in which both drugs would be taken in parallel by independent groups; to date, no such studies have been conducted.

It is also important to continue conducting studies of the effects of both drugs on specific groups of patients, which would allow greater individualization of treatment for the sake of specific and varying characteristics of individuals among all those suffering from an AUD.

## Figures and Tables

**Table 1:** The comparison of naltrexone and nalmafene.

Parameter	Naltrexone	Nalmefene
ATC Classification System	N07BB04	N07BB05
Medical indication	Treatment of alcohol use disorder (AUD) and opioids	Reductions in alcohol consumption in patients with AUD
Mechanism	Opioid receptor antagonist (μ, κ, δ); blocks the action of endorphins and opioids in the central and peripheral nervous system	Opioid receptor antagonist (μ, δ), κ receptor agonist; higher affinity for MOR, modifies reward system
Route of administration	Oral (PO), intramuscular (IM) (prolonged-acting injections)	Oral (PO), intravenous (IV), intramuscular (IM), subcutaneous (SC), intranasal (IN)
T_max_ (oral administration)	About 1 h	About 1.5 h
Protein binding	21%	About 30%
Main metabolizing enzymes	Aldo-ketoreductase 1C (mainly AKR1C4, to a lesser extent AKR1C1 and AKR1C2)	Bonding with glucuronic acid and sulfuric acid (glucuronidation, sulfation)
Metabolites	6-β-naltrexol, 2-hydroxy-3-methoxy-6-β-naltrexol	Nalmefene 3-O-glucuronide, nalmefene 3-O-sulfate, nornalmefene (further converted to nornalmefene 3-O-glucuronide and nornalmefene 3-O-sulfate)
Elimination	Mainly via kidney; 60% in 48 h as 6-β-naltrexol glucuronate and naltrexone hydrochloride	Mainly via kidney; 54% as nalmefene 3-O-glucuronide, other metabolites and nalmefene <3%
Half-life (t_1/2_)	PO: 3.9–10.3 h; 6-β-naltrexol: 13 h; IM: 5–10 days	PO: 12.5 h; IV: 10.8 h
Pharmacodynamic considerations	Prolongs abstinence, reduces alcohol consumption, reduces subjective enjoyment of alcohol	Reduces alcohol consumption by stopping the motivation to take alcohol, both spontaneous and withdrawal-induced
Structural information	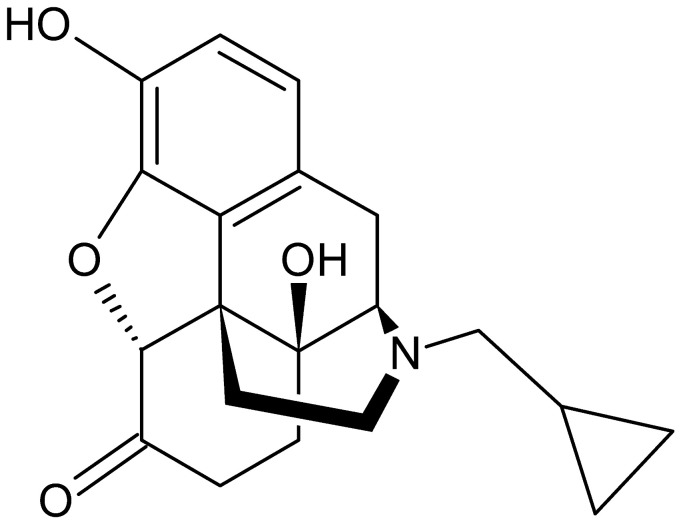	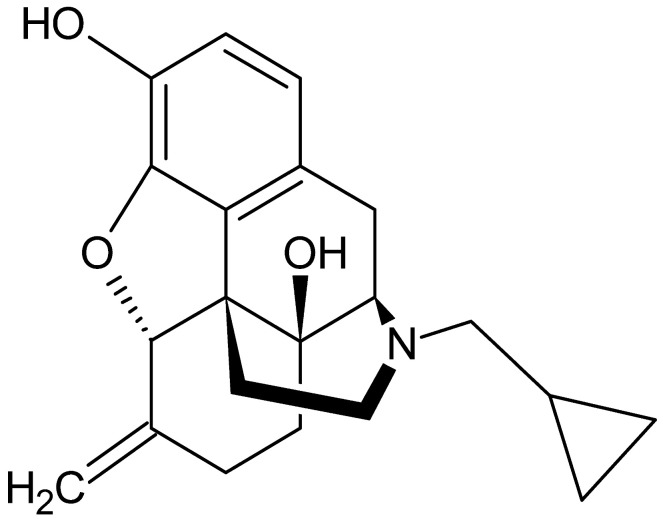

**Table 2:** Studies assessing the effect of nalmefene on AUD severity.

Authors	Mueller et al. [74]	Martín-Blanco et al. [75]	Di Nicola et al. [76]	Barrio et al. [77]
Type of study	exploratory data analysis, intervention trial, unblinded assessment	intervention trial, unblinded assessment, uncontrolled clinical trial	naturalistic study	Single arm observational study
Duration	12 weeks	8 weeks	24 weeks	1 year
Number of participants	45	25	65	110
Characteristics of treatment group	High DRL, elevated index of liver stiffness and/or hepatic steatosis	AUD with concomitant bipolar affective disorder	high DRL, 64.6% patients with comorbid psychiatric disease, 30.8% polyabusers	alcohol dependence diagnosis
Treatment (with 18 mg Nalmefene)	Orally as needed	Orally every morning	Orally as needed	Orally as needed
Decline of number of heavy drinking days (days/month)	13.5	4.40	6.68	13.5
Decline of transdermal alcohol concentration (g/day)	45.8	-	6.8	-
Liver tests	ALAT: 34.93 → 28.47 GGT: 72.51 → 60.28	Unmeasured	ALAT (non-comorbid): −23.7 ALAT (comorbid): −12 GGT (non-comorbid): −30.2 GGT (comorbid): −16.7	no statistically significant changes
Safety and tolerance (percent of patients with Treatment Emergent Adverse Event)	45%	20%	0.72 adverse effects per patient (most at the first dose)	26.4%

**Table 3:** Overview of selected clinical studies evaluating the efficacy of naltrexone in the treatment of alcohol use disorder.

Author (Type of Analysis)	Research Sample	Administration Form/Dosage	Duration	Main Endpoints	Key Results	Notes
Murphy et al. [78] (systematic review + meta-analysis)	1500 patients with moderate or severe AUD (DSM-IV)	LAI (150–400 mg IM monthly) + psychotherapy	2–6 months	DD, HDD, relapse risk	DD: WMD −2.0 [95% CI −3.4; −0.6] (*p* = 0.03); HDD: WMD −1.2 [95% CI −2.1; −0.2] (*p* = 0.02); no significant difference in relapse risk	Significantly better results than placebo in reducing DD and HDD
McPheeters et al. [79] (meta-analysis)	Moderate/severe AUD	LAI, PO 50 mg/d, PO 100 mg/d	12 weeks	DD, HDD	LAI: DD −4.99 [95% CI −9.49; 0.49]; HDD −4.68 [95% CI −8.63; −0.73]; PO 50 mg: DD −5.1 [95% CI −7.16; −3.04]; HDD −4.3 [95% CI −7.6; −0.91]; PO 100 mg: DD −2.3 [95% CI −5.6; 0.99]; HDD −3.1 [95% CI −5.8; −0.3]	PO 50 mg/d is the best studied and most effective form
Bernstein et al. (RCT *) [80]	244 patients	PO 50 mg/d vs. LAI	12 weeks	Adherence	PO: 37.9% vs. LAI: 62.5%	LAI more beneficial in groups with low adherence
Dermody et al. [82] (secondary analysis from MEMS)	56 patients	PO 50 mg/d	Several weeks	Daily life factors and adherence	Decrease in adherence after heavy drinking days; higher adherence with daily mobile self-assessments	Highlights need for strategies to resume treatment after relapse
Collins et al. (RCT) [85]	308 homeless individuals, 67% severe AUD	HaRT-A ± LAI NTX, HaRT-A ± placebo, TAU	12 weeks	SDPO **, DD/month	LAI: SDPO 12.81 (SD 3.30) vs. placebo 19.09 (SD 2.28); DD/month: 15.15 (SD 11.33) vs. 18.68 (SD 11.45)	Better outcomes with LAI + HaRT-A
Vutien et al. [86] (safety analysis)	Subgroup from Collins et al. study	LAI NTX vs. placebo	12 weeks	AST, ALT, GGT	No significant differences in enzyme changes between groups	LAI safe even in severe AUD
Miranda et al. [83] (RCT)	22 adolescents aged 15–19 years with AUD	PO 50 mg/d	8–10 days	Drinking propensity, binge risk	Drinking propensity ↓ 31% (*p* = 0.03), binge ↓ 54% (*p* = 0.003)	Preliminary evidence of efficacy in adolescents

Abbreviations explained: * RCT—randomized controlled trial; ** SDPO—standard drinks on peak occasion.

## Data Availability

Not applicable.

## Abbreviations

The following abbreviations are used in this manuscript:

AUD	Alcohol use disorder
TAC	total alcohol consumption
HDD	heavy drinking
WHO	World Health Organization
DALY-s	disability-adjusted life years
DSM-5	Diagnostic and Statistical Manual of Mental Disorders, 5th edition
GABA	γ-aminobutyric acid
VTA	ventral tegmental area
CNS	central nervous system
GPCRs	G protein-coupled receptors
GAD	glutamate decarboxylase
PAG	enzyme phospho-activated glutaminase
SSADH	Succinic semialdehyde dehydrogenase
DMS	dorsomedial striatum
MNS	medial striatal spiny neurons
GPe	globus pallidus
qPCR	quantitative real-time PCR
NMDAR	N-methyl-D-aspartate receptor
ETC	electron transport chain
mPTPs	mitochondrial permeability transition pores
DT	Delirium tremens
ROS	reactive oxygen species
DA	dopamine
NAc	nucleus accumbens
AWS	Alcohol Withdrawal Syndrome
ENKs	enkephalins
mPFC	medial prefrontal cortex
CeA	central nucleus of the amygdala
HPA	hypothalamic–pituitary–adrenal
ATC	Anatomical Therapeutic Chemical
AKR1C	aldo-keto reductase 1C
IV	intravenous
IM	intramuscular
SC	subcutaneous
IN	intranasal
DOA	duration of action
PO	Oral
DRL	Drinking Risk Level
TEAEs	treatment-emergent adverse events
GGT	γ-glutamyltransferase
ALAT	alanine aminotransferase
EMA	European Medicines Agency
DD	drinking days
HDDs	heavy drinking days
WMD	weighted mean difference
XR-NTX	Extended release naltrexone
HaRT-A	Harm Reduction Treatment for Alcohol
AST	aspartate aminotransferase
ALT	alanine aminotransferase
RCT	randomized controlled trial
SDPO	standard drinks on peak occasion
